# Immune Response to *Bifidobacterium bifidum* Strains Support Treg/Th17 Plasticity

**DOI:** 10.1371/journal.pone.0024776

**Published:** 2011-09-22

**Authors:** Patricia López, Irene González-Rodríguez, Miguel Gueimonde, Abelardo Margolles, Ana Suárez

**Affiliations:** 1 Immunology Area, Department of Functional Biology, University of Oviedo, Oviedo, Asturias, Spain; 2 Department of Microbiology and Biochemistry of Dairy Products, Instituto de Productos Lácteos de Asturias (IPLA), Consejo Superior de Investigaciones Científicas (CSIC), Villaviciosa, Asturias, Spain; Charité, Campus Benjamin Franklin, Germany

## Abstract

In this work we analyzed the immune activation properties of different *Bifidobacterium* strains in order to establish their ability as inductors of specific effector (Th) or regulatory (Treg) responses. First, we determined the cytokine pattern induced by 21 *Bifidobacterium* strains in peripheral blood mononuclear cells (PBMCs). Results showed that four *Bifidobacterium bifidum* strains showed the highest production of IL-17 as well as a poor secretion of IFNγ and TNFα, suggesting a Th17 profile whereas other *Bifidobacterium* strains exhibited a Th1-suggestive profile. Given the key role of Th17 subsets in mucosal defence, strains suggestive of Th17 responses and the putative Th1 *Bifidobacterium breve* BM12/11 were selected to stimulate dendritic cells (DC) to further determine their capability to induce the differentiation of naïve CD4^+^ lymphocytes toward different Th or Treg cells. All selected strains were able to induce phenotypic DC maturation, but showed differences in cytokine stimulation, DC treated with the putative Th17 strains displaying high IL-1β/IL-12 and low IL-12/IL-10 index, whereas BM12/11-DC exhibited the highest IL-12/IL-10 ratio. Differentiation of naïve lymphocytes confirmed Th1 polarization by BM12/11. Unexpectedly, any *B. bifidum* strain showed significant capability for Th17 generation, and they were able to generate functional Treg, thus suggesting differences between in vivo and vitro responses. In fact, activation of memory lymphocytes present in PBMCS with these bacteria, point out the presence *in vivo* of specific Th17 cells, supporting the plasticity of Treg/Th17 populations and the key role of commensal bacteria in mucosal tolerance and T cell reprogramming when needed.

## Introduction

Probiotics are live microorganisms that confer beneficial effects on health when supplied in adequate amounts [1], [2]. The genus *Bifidobacterium* is one of the most widely used probiotic bacteria, being predominant in breast fed infants [3]–[6].

It has been broadly accepted that probiotics, in addition to their role in the maintenance of the gastrointestinal barrier function [7]–[11], exhibit healthy properties through the immunomodulation of both mucosal and systemic immunity under healthy or pathogenic conditions [12], [13]. Gut colonization of bifidobacteria has been associated with some health-promoting effects, among which are reduction of certain harmful bacteria, alleviation or prevention of diarrhea, reduction of lactose intolerance, attenuation of inflammatory bowel disease symptoms, and relief of constipation [11]. Although the mechanisms underlying these benefits are not completely understood, in recent years, probiotics have raised interest due to their capability to regulate the immune response [12], [14] through their effects on intestinal mucosa dendritic cells (DCs). Gut DCs may sample antigens from food and bacteria of the intestinal lumen [15]–[17] and therefore could acquire a mature state and secrete specific cytokines [18]. In this respect, it has been described that distinct strains of *Bifidobacterium* can induce different maturation and cytokine production patterns on DCs in a strain-specific manner [18]–[21]. As a result, the different cytokine profiles released by DCs may direct the polarization of naïve CD4^+^ T cells towards different effector or regulatory T cell subsets [22], [23]. In fact, it has been described that different probiotics, such as *Lactobacillus plantarum* 299v, *Lactobacillus reuteri* DSM 12246, and *Lactobacillus johnsonii* La1, differ in their ability to induce T cell differentiation and cytokine production [24]–[27]. After the first studies, it became clear that certain probiotic strains cultured *in vitro* with immune cells induced IL-12 production, a key cytokine for promoting Th1 responses, characterized by the production of IFNγ and TNFα and essential for a successful defence against intracellular pathogen infections [28]. On the contrary, other strains induced high amounts of the anti-inflammatory cytokine IL-10, generating a Th2 profile. However, the classic Th1/Th2 division was later changed after the characterization of Th17 cells. This population produces IL-17, the signature effector cytokine for this subset, and presents critical functions in host defence against extracellular bacterial and fungal pathogens, particularly those encountered at mucosal surfaces [29]–[36]. Additionally, the gut immune system has diverse mechanisms of tolerance that avoid uncontrolled Th1, Th2 or Th17 effector responses to self and intraluminal antigens. Particularly relevant among them is the action of CD25^high^ FOXP3^+^ regulatory T cells, which act by suppressing effector responses [37]. These cells are derived from the thymus (Treg), or may be induced in peripheral organs (iTreg), including the gut mucosa [38]. Moreover, although there are few works reporting bacterial iTreg induction, there is increasing evidence that some probiotic bacteria might induce FOXP3^+^ regulatory T cells from naïve precursors [27], [39]–[41]. The generation of iTreg cells by probiotics could have a beneficial effect on allergy and autoimmune diseases, whereas induction of Th1 or Th17 effector cells would be more interesting in the defense against pathogens. Consequently, to know the specific immunomodulatory ability of each strain could allow us to select specific probiotic bacteria for further biotechnological or clinical applications in function of the final requirement. Given the need for appropriate *in vitro* studies to characterize the immunomodulatory capacity of different probiotic strains before their use, in this study, we analyzed the specific immune activation properties of 21 different *Bifidobacterium* strains in order to establish their ability as inductors of effector or regulatory T cell responses.

## Results

### *Bifidobacterium* spp. show different ability to induce cytokine production by immune cells

**Figure 1:**
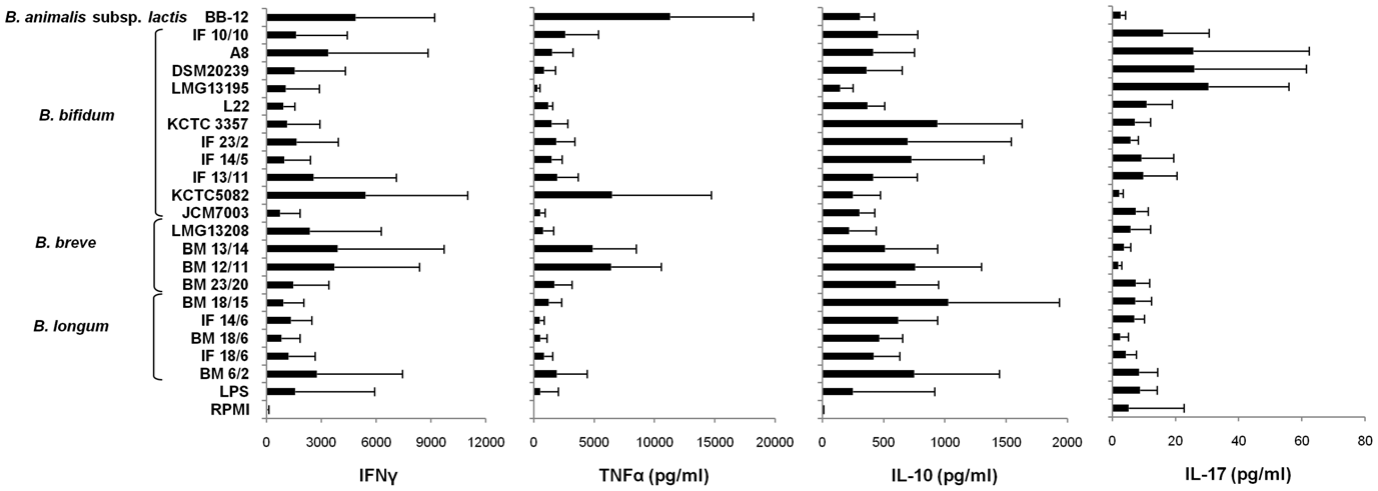
Cytokine production by human PBMC stimulated with different bifidobacteria strains. PBMCs were cultured with RPMI alone, or in the presence of LPS (*E. coli*) or 21 UV-killed bifidobacteria strains at bacteria:cell ratio of 5∶1. After four days of culture, supernatants were collected to quantify the amounts of IL-10, IL-17, IFNγ and TNFα by a multiplex assay (Flex set CBA). Histogram shows mean and standard deviation obtained in four independent experiments performed with different blood donors.

*Bifidobacterium* strains are commensal microorganisms usually present in the human gut, where they interact with immune cells [42]. To study the effect that these interactions may have in modifying cytokine production, we cultured PBMCs isolated from healthy individuals with 21 different UV-killed *Bifidobacterium* strains (bacteria:cell ratio 5∶1) and the amount of Th1 (IFNγ, TNFα), Th2 (IL-10) and Th17 (IL-17) cytokines was determined in the supernatants after 4 days of culture by a multiplex immunoassay. As can be observed in Figure 1, no relevant cytokine production was detected in unexposed PBMCs (RPMI), whereas LPS (from *E. coli*) treatment and the whole set of *Bifidobacterium* strains tested induced cytokine production, although clear differences in the levels of IL-17, IL-10, TNFα and IFNγ (p = 0.001, p = 0.002, p<0.0001 and p = 0.001, respectively; Kendall test) induced by different strains were observed. Specifically, *B. bifidum* IF10/10, A8, DSM20239 and LMG13195 strains showed the highest production of IL-17, existing significant differences compared with the rest of bifidobacteria analyzed (p = 0.014, T-test). In addition, they induced relatively poor secretion of IFNγ and TNFα, thus suggesting a Th17 profile. On the contrary, the strains *B. animalis* subsp. *lactis* BB-12, *B. breve* BM12/11, BM13/14 and *B. bifidum* KCTC5082 exhibited the highest capacities for TNFα (p = 0.011, T-test) and IFNγ production (p = 0.028, T-test) compared with the other tested bacteria, a cytokine profile suggestive of a Th1 polarization. Also, *B. bifidum* KCTC3357, IF23/2, IF14/5 and *B. longum* BM18/15 showed the higher production of IL-10, as well as low levels of Th1 and Th17 cytokines (p = 0.036, T-test).

These results indicate that bifidobacteria can stimulate immune cells to produce different cytokines in a strain-specific manner.

### *Bifidobacterium* spp. induce different T cell polarizing ability on DCs

**Figure 2:**
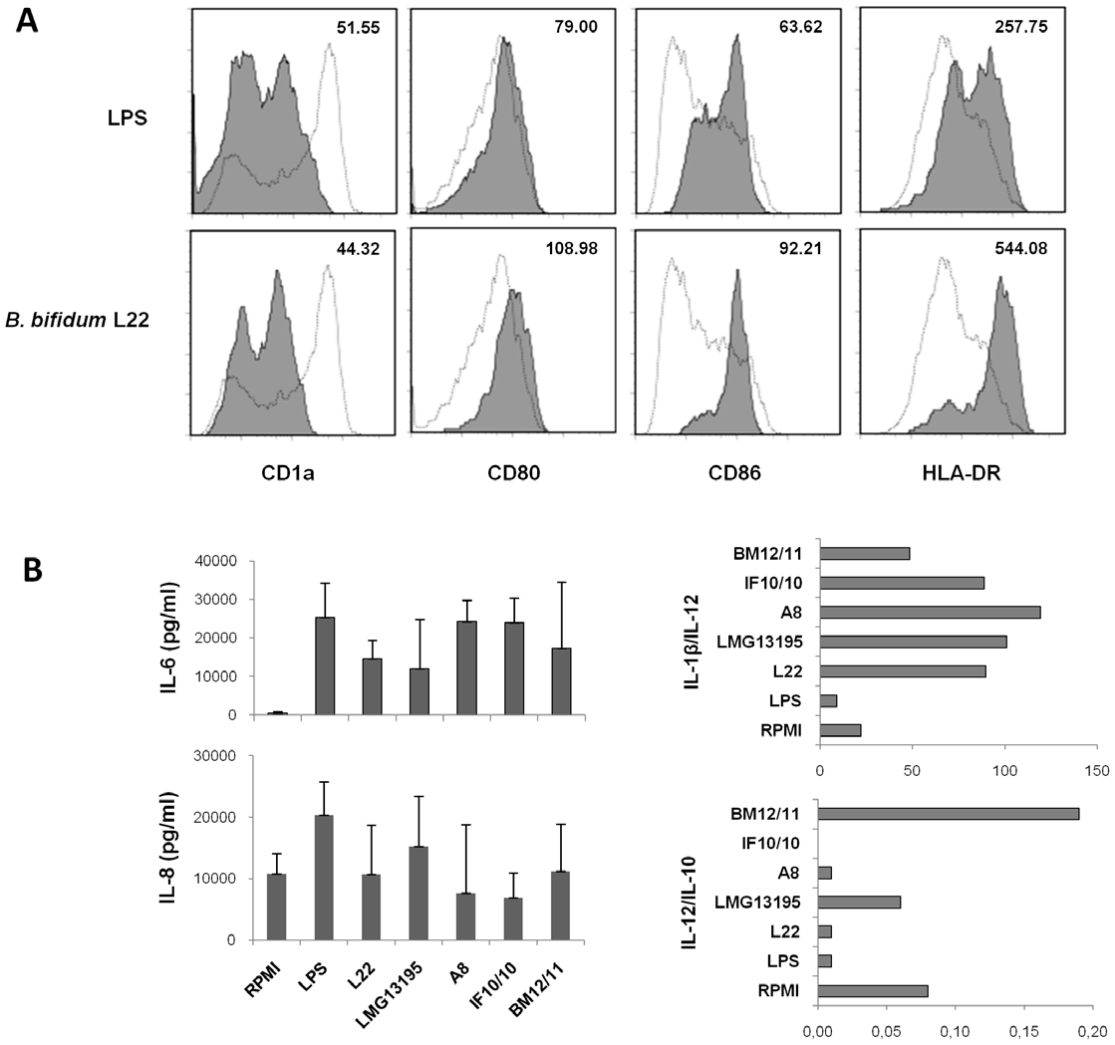
Maturation and cytokine secretion of monocyte-derived DC treated with *Bifidobacterium* strains. Immature DC were cultured for 48 h with lipopolysaccharide (LPS) as maturation control, or with different bifidobacterial strains at a bacteria:cell ratio of 10∶1. (A) Expression of CD1a, CD80, CD86 and HLA-DR was analyzed by flow cytometry. Representative profile after 48 hours of culture with LPS or bifidobacteria strain (*B. bifidum* L22). Empty histograms belong to immature DCs (discontinuous line), and solid histograms represent specific staining of the indicated cell surface marker in LPS or *Bifidobacterium*-stimulated DC. Mean fluorescence intensities (MFI) obtained after subtracting background staining are provided in each histogram. (B) DC cytokine profile induced by exposure to *Bifidobacterium* strains. The concentration of IL-8, IL-6, IL-1β, IL-10 and IL-12 was determined using a multiplex assay. Independent ratios were calculated from each donor. Data are expressed as mean and standard deviation of four independent experiments performed with different blood donors.

The different Th subsets present in PBMCs are the result of the adaptative immune response to specific antigens, which has been previously endocyted, processed and presented by DCs to naïve CD4^+^ T cells. Subsequently, we wanted to determine the capacity of DCs treated with specific *Bifidobacterium* strains to differentiate CD4^+^ naïve T lymphocytes towards different effector Th cells. Since it has been described that Th17 cells are implicated in the defence of mucosal barrier against extracellular pathogens, we found it especially interesting to know the effect of *B. bifidum* in the possible induction of this subset. For this purpose, we selected four *Bifidobacterium* strains previously found to be suggestive of a Th17 differentiation (*B. bifidum* LMG13195, L22, A8, IF10/10) and a probable Th1-inductor strain (*B. breve* BM12/11) as control. Monocyte-derived DCs (inmature DC) were exposed to UV-killed bacteria (bacteria:cell ratio 10∶1), or to LPS, and after 48 hours DC phenotype and cytokine production were evaluated as indicators of DC maturation. The maturation pattern of DCs was assessed by flow cytometric analysis of surface marker expression. All *Bifidobacterium* strains tested were able to up-regulate the expression of HLA-DR and the costimulatory molecules CD80 and CD86, and to down-regulate the production of CD1a at similar or even higher levels than those observed with LPS treatment, used as reference for *in vitro* DC maturation (Figure 2A shows a representative example), thus indicating their mature phenotype.

Next, we analyzed the amount of IL-6, IL-8, IL-1β, IL-12p70 and IL-10 present in culture supernatants of bifidobacteria treated DCs (Figure 2B). All strains induced the production of the proinflammatory cytokines IL-6 and IL-8 in a similar or lower level than LPS treatment. In an attempt to predict subsequently DC-mediated CD4^+^ T cell differentiation, we calculated the cytokine ratios relevant for Th17 (IL-1β/IL-12) and Th1 (IL-12/IL-10) cell polarization. Thus, *B. bifidum* LMG13195, L22, A8 and IF10/10 exhibit high IL-1β/IL-12 and low IL-12/IL-10 index, data compatible with the cytokine pattern exhibited by PBMCs treated with these bacteria. Conversely, the highest IL-12/IL-10 ratio was presented by *B. breve* BM12/11, in accordance with results from PBMCs cultures. All these data suggest that different analyzed bifidobacteria strains are able to generate mature DCs prone to induce Th17 or Th1 effector lymphocytes.

**Figure 3:**
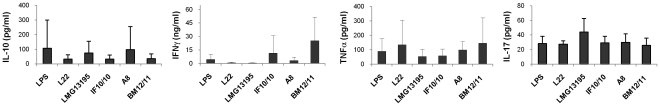
Cytokine production by CD4^+^ T lymphocytes stimulated with *Bifidobacterium* treated DCs. DCs maturated with LPS, *B. bifidum* LMG13195, L22, A8 and IF10/10 or *B. breve* BM12/11 were cocultured with allogeneic naïve CD4^+^CD45RA^+^ T cells at T-cell:DC ratio of 1∶10. The concentration of IL-10, IL-17, IFNγ and TNFα was quantified by multiplex assay. Bars represent the mean and standard deviation of four independent experiments performed with different blood donors.

Then, to confirm this hypothesis, we determined the ability of *Bifidobacterium*-DCs to generate specific Th subsets (Th1/Th2/Th17) from naïve CD4^+^ lymphocytes. To this end, DCs maturated with LPS or with the selected strains were cocultured with highly purified allogenic naïve CD4^+^CD45RA^+^ T cells at T-cell:DC ratio of 1∶10, and cytokine levels were quantified after 12 days of culture (Figure 3). All DC-stimulated CD4^+^ lymphocytes showed poor secretion of IL-10 whereas *B. breve* BM12/11-DCs induced the highest levels of IFNγ and relatively high amounts of TNFα, as expected from our previous results. However, although DCs treated with *B. bifidum* LMG13195 were the most efficient at inducing IL-17 production, no significant differences were found among the strains. These results suggest that the *B. bifidum* strains previously selected by their putative Th17 profile were not able to induce this differentiation from naïve T cells, at least in our *in vitro* conditions, whereas *B. breve* BM12/11 can efficiently promote the generation of Th1 responses.

### *B. bifidum*-DCs promote the generation of functional Treg cells

**Figure 4:**
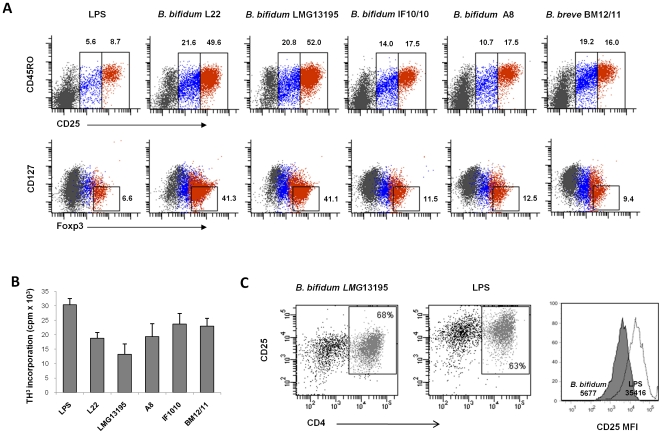
Induction of functional Treg cells by *Bifidobacterium* strains. (A) CD4^+^CD45RA^+^ T cells were co-cultured with DCs stimulated with different bifidobacterial strains in a 1∶10 ratio. After 12 days of culture determination of Treg cells was assessed by extra/intra-cellular staining by flow cytometry. Different gates for CD45RO^+^ cells with low/intermediate (blue dots) and high (red dots) expression of CD25 were defined, allowing the quantification of CD25^high^ FOXP3^high^CD127^low^ lymphocytes. (B) PHA-activated effector cells (allogenic PBMCs) were cultured in the presence of cells obtained after 7-day stimulation of PBMCs with LPS or *Bifidobacterium* spp. After 4 days of culture, cells were pulsed with [^3^H]thymidine and incorporation was measured 16 h later. Histogram shows mean and standard deviation of the cpm in stimulated triplicates obtained in 4 independent experiments performed with different blood donors. (C) Analysis of CD4/CD25 expression in the same cultures.

In view of these inconclusive results, we wanted to determine the possible effect of these bacterial strains on the induction of regulatory T cells (Treg). To this end, we analyzed the regulatory phenotype of the cells obtained after 12 days of stimulation of purified naïve CD4^+^ lymphocytes (CD45RA^+^) with *Bifidobacterium*-DCs and determined their ability to suppress the proliferation of activated effector cells. Although no definitive surface markers for human Treg have been found, it has been described that only CD4^+^CD45RO^+^ cells (memory/effector phenotype) expressing high levels of CD25 and Foxp3 are able to efficiently suppress proliferative responses, whereas low/intermediate expression of these two markers are characteristic of activated cells [43]. Therefore, to analyze the Treg population we quantified, by flow cytometry, the intracellular expression of FOXP3 and surface levels of CD127, a marker lost in Treg cells, in CD45RO^+^ lymphocytes presenting low and high CD25 levels, according to the regions shown in Figure 4A. Interestingly, naïve CD4^+^ T cells stimulated with all *Bifidobacterium*-DCs presented a higher percentage of CD25^high^ and CD25^low^ CD45RO^+^ cells than lymphocytes cocultured with LPS-DCs, thus indicating a higher immune stimulating ability. These figures, however, differed among the tested strains, *B. bifidum* 13195-DCs and *B. bifidum* L22-DCs being the most efficient up-regulators of CD25 expression. Moreover, they were the highest inducers of Foxp3 expression, thus generating a considerable amount of CD25^high^FOXP3^high^CD127^low^ cells, a phenotype characteristic of Treg cells (Figure 4A).

Finally, we investigated the regulatory function of these putative Treg lymphocytes by assessing their ability to suppress the proliferation of effector cells, the typical function of regulatory T cells. To this end, we determined the capability of these possible suppressor cells, obtained after stimulation of PBMC with LPS or *Bifidobacterium* spp., to inhibit the proliferation of allogenic PBMC stimulated with PHA (effector cells). Figure 4B shows that cells stimulated with *B. bifidum* LMG13195 efficiently suppress the proliferation of effector cells. This effect was not due to cell death, since analysis of CD4 and CD25 expression in PHA-stimulated effector cells co-cultured with *B. bifidum* treated cells did not cause a reduction in the amount of CD4^+^ lymphocytes compared with cells treated with LPS (Figure 4C). Moreover, we were able to observe a significant lower expression of CD25, indicative of a low activated state of effector cells, in accordance with their reduced proliferation rate.

## Discussion

The interest in the immunomodulatory properties of probiotic bacteria derives from the observation that intestinal microbiota is critical for development of the immune system [44]. Since it is known that different probiotic bacteria present specific immunoregulatory effects, it appears reasonable to assume that changing the microbiota with the use of well-studied probiotics may influence the immune system in a desired direction.

In the present work we demonstrated great differences between *Bifidobacterium* strains in the ability to activate DCs and to drive the differentiation of naïve T cells. Firstly, in order to obtain an approximation in the type of Th cell they could promote, we analyzed the effect of 21 *Bifidobacterium* strains, on the induction of Th1 (IFNγ, TNFα), Th2 (IL-10) and Th17 (IL-17) cytokines by human PBMCs. We found that different bifidobacteria stimulate immune cells to produce strain-specific cytokines, suggesting that they could possess different T cell polarizing abilities. Thus, cells stimulated with strains belonging to *B. animalis* subsp. *lactis* (BB-12), *B. breve* (BM12/11, BM13/14) and *B. bifidum* (KCTC5082) exhibited the highest levels of TNFα and IFNγ, representative of a Th1 response. On the other hand, some *B. bifidum* stains, such as IF10/10, A8, DSM20239 and LMG13195, showed a significant enhancement of IL-17, as well as a poor secretion of IFNγ and TNFα, suggesting a possible Th17 profile.

Immune stimulating activity of the majority of probiotics commonly added into functional foods is mediated by promoting Th1 responses. However, Th17 cells are critical to enhance host protection against extracellular bacteria and fungi, which are not efficiently cleared by Th1 and Th2 responses [29], [31]. Thus, on the basis of our results, we considered of interest the selection of Th17-inducer strains for further evaluation of their possible biotechnological use.

Since mature DCs play a key role in T cell function, leading to proliferation and differentiation towards effector (Th) or regulatory (Treg) cells, we analyzed the ability of DCs treated with the possible Th17-inducer *B. bifidum* strains for the generation of Th17 cells. Accordingly with previous works [19], [25], [45]–[47], all *Bifidobacterium* strains tested were able to maturate DCs, however different patterns of cytokine production were detected. Therefore, differences in the induction of IL-12, IL-10, and IL-1β by DCs could be an indicator of the type of Th response that they may promote. IL-12 is a key immunoregulator cytokine with a central role in promoting Th1 responses, essential for a successful defence against intracellular pathogen infections [28]. On the other hand, the anti-inflammatory cytokine IL-10 exerts an opposite immunoregulatory effect, suppressing the production of IL-12 and Th1 cytokines and it may direct CD4^+^ T cells towards Th2 cell differentiation [48]. In addition, recent studies have provided evidence that IL-1β upregulates the expression of the transcription factor RORCv2 which can drive human Th17 generation [49]–[51]. Our results showed that DCs exposed to the possible Th17-inducer *B. bifidum* strains exhibited high IL-1β/IL-12 and low IL-12/IL-10 proportions, a profile compatible with a Th17 cell differentiation. Conversely, the highest IL-12/IL-10 ratio was presented by *B. breve* BM12/11, suggesting that it could promote the induction of Th1 responses. These results support previous studies showing that probiotic bacteria influence DC function in a strain-specific manner [46], [52].

Finally, we analyzed the ability of DCs treated with the selected Th1 and Th17 strains to differentiate naïve CD4^+^ T lymphocytes into effector or regulatory cells. As expected, *B. breve* BM12/11-DCs induced the highest levels of TNFα and IFNγ, but, surprisingly, although *B. bifidum* LMG13195-DCs upregulated IL-17 production, no significant differences were found compared with the other strains, suggesting that they were unable to generate Th17 cells from naïve T lymphocytes in these *in vitro* conditions. In this regard, it is known that the development of Th17 cells is closely linked to the generation of iTreg, since both processes share common cytokine signaling pathways [30], [36], [53]. Moreover, several studies revealed the plasticity in the polarization and balance between Treg and Th17 cell subsets coexisting in the same tissues [54], [55]. Besides, it has been described that FOXP3^+^ Treg cells can secrete IL-17 and express high levels of RORγt, as well as exert suppressor functions [56]–[58], suggesting that human Th17 cells could originate from Treg subsets. Indeed, Esposito *et al* [58] hypothesize that CD4^+^FOXP3^+^RORγt^+^ T cells represent a transient population, able to create both Th17 and Tregs.

In view of these results, we next studied the effect of the selected strains on the induction of Treg cells. This population is primarily characterized by the presence of high levels of CD25 and low expression of CD127. However, the transcription factor FOXP3 is considered the best functional marker of Tregs, although it is also transiently expressed in recently activated non-Treg CD4^+^ lymphocytes [59], [60]. Interestingly, our results showed that DCs maturated with the bifidobacteria tested presented a higher ability to activate naïve T cells than standard LPS-DCs, since they induced a higher proportion of CD45RO^+^CD25^+^ cells. However, only DCs treated with *B. bifidum* LMG13195 and L22 generated a high proportion of CD25^high^FOXP3^high^CD127^low^ cells. The expression of FOXP3 was quantified after 12 days in culture, which is far longer than the transient up-regulation of this molecule observed in activated effector cells [60], [61]. In addition, *in vitro* functional assays showed that Treg cells induced by *B. bifidum* LMG13195 were able to suppress the proliferation of effector cells more efficiently than the other strains analyzed. In this regard, Kwon *et al* recently observed that administration of the probiotic mixture ITR5 results in an enhancement of the CD4^+^FOXP3^+^ Treg population, and suppression of the progression of murine experimental inflammatory bowel disease, atopic dermatitis and rheumatoid arthritis [62]. Similarly, oral administration of a probiotic combination exerted a therapeutic effect on experimental autoimmune encephalomyelitis mediated by Treg [63]. However, the possible relationship with IL-17 production has not been determined.

The lamina propia of the small intestine contains large numbers of Th17 and Treg cells [30], [36], [53], two populations implicated in antagonistic functions. Tregs have a central homeostatic role in avoiding autoimmunity and allergy. However, the presence of mechanisms that rapidly block their suppressive activity and facilitate immune activation during an acute microbial infection are necessary. In this situation, diverse cell types secreted inflammatory cytokines generating a proinflammatory environment to clear the infection. In this regard, recent studies have indicated that Treg cells possess the capacity to produce IL-17 when they are activated in the presence of the proinflamatory cytokines IL-1β and IL-6, presenting sustained FOXP3 expression and a reversible loss of suppressive function [56], [64], [65]. Actually, this Th17/Treg plasticity could explain our apparently discordant *in vitro* and *ex vivo* results. DCs matured *in vitro* with specific *Bifidobacterium* strains polarize naïve T lymphocytes towards regulatory FOXP3^+^ cells, whereas in *ex vivo* experiments the same commensal bacteria induce the production of IL-17, probably due to the activation of memory Th17 cells present in PBMCs from healthy individuals. These data support that, *in vivo*, some commensal bacteria could maintain mucosal tolerance through the generation of a Treg pool, but, under danger signals or inflammatory stimulation, these cells could transdifferentiate to a Th17 effector subset, relevant in the defense against extracellular pathogens. In fact, a key role has been reported for commensal microbiota in the development of both Th17 and Treg cells [32], [66], [67].

**In summary**, the present work demonstrates great differences between *Bifidobacterium* strains in their ability to activate DCs and to drive the differentiation of naïve T cells. Our results support the fact that specific food and commensal bacteria may play a role in balancing the development of Treg and Th17 cell compartments in the intestine through the existence of Treg cells with plasticity to show an effector function, secreting IL-17, or a regulatory action, suppressing activation of the immune system, depending on the environment and the nature of the stimuli [32]. For instance, colonization of the gut by bacteria able to favour a Treg polarization, such as *B. bifidum* LMG13195, represents an attractive goal in the prevention and treatment of inflammatory diseases characterized by an overreaction of the immune system, such as autoimmune diseases, asthma and allergy. Moreover, since Treg cells may be capable of IL-17 secretion under certain conditions, adaptive immune responses against mucosal extracellular pathogens cannot be impaired.

## Materials and Methods

### Ethics statement

Ethics approval for this study was obtained from the Bioethics Committee of CSIC (*Consejo Superior de Investigaciones Científicas*) and from the Regional Ethics Committee for Clinical Research (*Servicio de Salud del Principado de Asturias*). All determinations were performed with fully informed written consent from all participants involved in the study.

### Bacterial strains and culture conditions

The different bacterial strains used in this study are shown in Table 1. All cultures were incubated at 37°C in anaerobic conditions (10% H_2_, 10% CO_2_ and 80% N_2_) in a chamber Mac 500 (Don Whitley Scientific, West Yorkshire, UK). For stimulation of immune cell experiments, overnight bacterial cultures were used to inoculate (1%) 150 ml of IMDM broth (Sigma Chemical Co, St. Louis, MO, USA, reference number I6529) supplemented with the following compounds: 16.5 mM cysteine, 74.0 µM adenine, 89.2 µM uracil, 65.7 µM xanthine, 66.2 µM guanine, 2.47 mM ammonium citrate tribasic, 12.19 mM sodium acetate, 166 µM MnSO_4_•H_2_O, 17.4 µM ZnSO_4_•7 H_2_O, 10.5 µM CoCl_2_•6 H_2_O, 0.4 µM CuSO_4_•5 H_2_O, 25.2 µM FeCl_2_•4 H_2_O, 22.2 mM fructose, 22.2 mM glucose, 0.2% of Tween 80 and 1% of the permeates resulting from the dialysis (2 kDa cut-off membrane) of 10 times concentrated MRS broth (Difco Laboratories, Detroit, MI, USA) dialyzed against 10 volumes of milli-Q water. After incubation, cultures were harvested by centrifugation, bacterial pellets were washed three times in PBS buffer (Oxoid Limited, Hampshire, UK) and resuspended in 5 ml of the same buffer. Bacterial levels in the cell suspensions were determined by plate counting and cultures were killed by exposing them to UV light in a UV chamber (15 W, Selecta, Barcelona, Spain) for 90 min. Plate counting was carried out after UV treatment to corroborate the absence of bacteria able to recover in the proper medium. UV treated bacterial suspensions were then distributed in single use aliquots, frozen in liquid N_2_ and stored at −80°C until use.

**Table 1:** Bacterial strains, origin and relevant phenotype.

Strain	Origin	Relevant characteristics	Selected reference
*B. animalis* subsp. *lactis* BB-12	Intestine of adult	Known probiotic strain	[68]
*B. longum* BM 18/6 (IPLA 20002)	Breast milk	-	[6]
*B. longum* IF 18/6 (IPLA 20023)	Infant feces	-	This work
*B. longum* BM 6/2 (IPLA 20001)	Breast milk	-	[6]
*B. longum* BM 18/15 (IPLA 20003)	Breast milk	Resistant to bile	[6]
*B. longum* IF 14/6 (IPLA 20011)	Infant feces	*-*	This work
*B. bifidum* IF 10/10 (IPLA 20015)	Infant feces	-	[19]
*B. bifidum* A8	Dairy product	Mucin-degrading strain	[69]
*B. bifidum* DSM20239	Adult	Mucin-degrading strain	[70]
*B. bifidum* LMG13195	Infant, intestine	-	[71]
*B. bifidum* L22	Adult	Mucin-degrading strain	[70]
*B. bifidum* KCTC3357	Human feces	-	-
*B. bifidum* IF 23/2 (IPLA 20017)	Infant feces	-	This work
*B. bifidum* IF 14/5 (IPLA 20024)	Infant feces	-	This work
*B. bifidum* IF 13/11 (IPLA 20016)	Infant feces	-	This work
*B. bifidum* KCTC5082	-	-	-
*B. bifidum* JCM7003	Human feces	-	-
*B. breve* LMG13208	Infant	*In vitro* antimutagenic activity	[72]
*B. breve* BM 13/14 (IPLA 20005)	Breast milk	-	[6]
*B. breve* BM 12/11 (IPLA 20004)	Breast milk	*-*	[6]
*B. breve* BM 23/20 (IPLA 20006)	Breast milk	Tolerant to gastric juice	[6]

DSM, German Collection of Microorganisms and Cell Cultures; LMG/BCCM, Belgian Co-ordinated Collections of Microorganims; KCTC, Korean Collection for Type Cultures; JCM, Japan Collection of Microorganisms.

### Isolation of PBMCs

Human peripheral blood mononuclear cells (PBMCs) were obtained from standard buffy-coat preparations from routine blood donors (Asturian Blood Transfusion Center, Oviedo, Spain) by centrifugation over Ficoll-Hypaque gradients (Lymphoprep, Nycomed, Oslo, Norway). All blood donors (the number is specified in each figure legend) were healthy adult volunteers without any pathology or treatment.

### PBMCs stimulation with *Bifidobacterium* strains

2×10^4^ PBMCs were incubated with bacteria at PBMCs:bacteria ratio of 1∶5. All cultures were performed in triplicate wells in 200 µl of complete RPMI medium (RPMI 1640 containing 2 mM L-glutamine and 25 mM Hepes, Bio Whitaker, Verviers, Belgium, supplemented with 10% heat-inactivated fetal calf serum and the antibiotics streptomycin and ampicillin at 100 µg/ml) at 37°C and 5% carbon dioxide. After 4 days of culture, 100 µl of supernatants from these cultures were collected for cytokine quantification.

### Generation of Monocyte-derived DCs

Monocytes were isolated from previously obtained PBMCs by negative selection using the Human Monocyte enrichment kit, according to the protocol provided by EasySep, StemCell Technologies (Canada). Purified monocytes were >95% CD14^+^. Immature DCs were obtained from isolated monocytes by standard procedures. Thus, monocytes were cultured in 24-well plates at a concentration of 5×10^5^ cells/ml for 7 days at 37°C and 5% carbon dioxide in complete RPMI medium in the presence of recombinant human (rh) IL-4 (35 ng/ml) and rhGM-CSF (70 ng/ml) (R&D Systems, Abingdon, UK). At days 2 and 5, 0.5 ml of the medium was removed without disturbing the clusters of developing DC and 0.5 ml of freshly made GM-CSF- and IL-4-containing medium was added to the wells, restoring the final volume in each well to 1 ml. At day 7, immature DCs were recovered, washed and resuspended in complete RPMI medium at 5×10^5^ cells/ml for subsequent maturation.

### Stimulation of monocyte-derived DCs with bifidobacteria

To examine the effects of bifidobacteria on DC maturation, different *Bifidobacterium* strains, killed by UV radiation, were added to immature DCs at a DC:bacteria ratio of 1∶10 in complete RPMI medium. Parallel cultures were treated with 1 µg/ml LPS from *E. coli* 0111:B4 (Sigma), as a positive control of maturation. After 48 hours, supernatants from these cultures were collected, clarified by centrifugation and stored at −20°C for cytokine analysis whereas DCs were harvested for phenotypic characterization.

### Flow cytometric analysis

Phenotypic studies of DCs, T lymphocytes and PBMCs were performed after staining with the appropriate monoclonal antibody (mAb) using a FACSCanto II flow cytometer (Becton Dickinson, BD Biosciences, San Diego, CA). DCs were stained with anti-CD86 fluorescein isothiocyanate (FITC), CD80 phycoerythrin (PE), HLA-DR (PE-Cy5) mAb, anti- CD1a-FITC mAb, or with the corresponding isotype matched conjugated irrelevant mAb as a negative control. All mAb were supplied by Becton Dickinson Pharmingen. Staining was performed for 30 min at 4°C, and cells were washed twice in staining buffer and resuspended in PBS. To determine Treg phenotype, cultured CD4+ lymphocytes were first extracellularly stained with CD45RO-APC, CD25-FICT and CD127 PE-Cy7 (eBiosciences, San Diego, CA). Then, cells were fixed, permeabilized and intracellularly stained with anti-FOXP3 PE (clone PCH101) following the manufacturer's instructions (eBiosciences). Isotype controls were used to set up quadrants and CD4^+^CD25^+^ T cells were subdivided into CD25^low^ and CD25^high^ populations. A minimum of 10,000 cells were acquired and analyzed using the FACSDiva Software 6.1.2 (BD Biosciences). The specific fluorescence intensity was quantified as the mean fluorescence intensity (MFI) calculated by subtracting the background of isotype-matched control staining from the total fluorescence.

### Naïve CD4+ T cell stimulation with *Bifidobacterium*-DCs

CD4+ T cells were isolated from PBMCs by negative selection using the Human CD4+ T Cell Enrichment Kit (EasySep, Stem Cell Technologies Inc.), following manufacturer's instructions, and then naïve CD45RA^+^ T cells were separated after depletion of CD45RO+ cells (Miltenyi Biotec GmbH, Bergisch Gladbach, Germany). Monocyte-derived DCs maturated after 48 h treatment with LPS or with *B. bifidum* LMG13195, L22, A8, IF10/10 or *B. breve* BM12/11 (*Bifidobacterium*-DCs) were cocultured with purified allogeneic CD4^+^CD45RA^+^ T cells in 48-well plates at DC:T-cell ratio of 1∶10. At day 5 and 8, cells incubated with all treatments were expanded with IL-2 (30 U). After 12 days, culture supernatants were collected for cytokine measurements, and cells were used to analyze the Treg phenotype by flow cytometry.

### Suppression assays

The suppressive function of *Bifidobacterium*-stimulated PBMCs was assessed by determining their ability to inhibit effector cell proliferation by quantifying [^3^H]thymidine incorporation in cultured cells. To this end, 10^5^ PBMCs stimulated with LPS or different *Bifidobacterium* strains were cultured with PBMCs activated with 2 µg/ml PHA (effector cells) at ratio 1∶1. All cultures were performed in triplicate wells in 200 µL of complete medium in 96-well round-bottom microtiter plates (Costar, Cambridge, MA). At day 4 of culture, 1 µCi/well [^3^H]thymidine was added to the cultures. After 16 h cells were harvested onto glass fiber filters for counting cell-incorporated [^3^H]thymidine using standard scintillation techniques (Packard Instruments, Downers Grove, IL). Results were determined as the mean counts per minute (cpm) values measured in stimulated triplicates.

### Cytokine determination

Cytokine levels in cell culture supernatants were quantified by a multiplex immunoassay (cytometric bead array, CBA, BD) using FacsCantoII flow cytometer (BD). To determine cytokine production of stimulated DCs the Human Inflammation kit for CBA was used (IL-8, IL-6, IL-1β, IL-10, TNFα and IL-12p70) whereas quantification of cytokine production by cultured CD4+ T lymphocytes and PBMCs was performed using a Flex Set for CBA including IL-17, IL-10, TNFα and IFNγ. Briefly, microbeads with distinct fluorescence intensities, coated with captured antibody specific for each cytokine, were incubated with serum samples and PE-conjugated detection antibodies for 2 hours. The samples were then washed, resuspended in 300 µl wash buffer, and analyzed using FCAP array software (BD Biosciences). The detection limits were: IL-8: 3.6 pg/ml; IL-1β: 7.2 pg/ml; IL-6: 2.5 pg/ml; IL-10: 3.3 pg/ml; TNFα: 3.7 pg/ml and IL-12p70: 1.9 pg/ml for the Human Inflammation kit and IL-17: 0.3 pg/ml; IL-10: 0.13 pg/ml; TNFα: 0.7 pg/ml and IFNγ: 0.8 pg/ml for the Flex Set.

### Statistical analysis

The Kolmogorov–Smirnov test was used to assess the normal distribution of the data. Cytokine levels were normally distributed and then differences between cell treatments were evaluated by the parametric T-test. Due to the existence of genetically determined differences between individuals in cytokine production, Kendall W tests for related samples were used. Results were represented by mean ± standard deviation. The SPSS 18.0 statistical software package (SPSS Inc., Chicago, IL) was used for all determinations and a value of p<0.05 was considered significant.
